# High-Dose Dexamethasone Versus Tocilizumab in Moderate to Severe COVID-19 Pneumonia: A Randomized Controlled Trial

**DOI:** 10.7759/cureus.20353

**Published:** 2021-12-11

**Authors:** Naveen B Naik, Goverdhan D Puri, Kamal Kajal, Varun Mahajan, Ashish Bhalla, Sandeep Kataria, Karan Singla, Pritam Panigrahi, Ajay Singh, Michelle Lazar, Anjuman Chander, Venkata Ganesh, Amarjyoti Hazarika, Vikas Suri, Manoj K Goyal, Vijayant Kumar Pandey, Narender Kaloria, Tanvir Samra, Kulbhushan Saini, Shiv L Soni

**Affiliations:** 1 Anesthesia and Intensive Care, Postgraduate Institute of Medical Education and Research, Chandigarh, IND; 2 Internal Medicine, Postgraduate Institute of Medical Education and Research, Chandigarh, IND; 3 Anesthesia, BronxCare Health System, New York, USA; 4 Anesthesia, Postgraduate Institute of Medical Education and Research, Chandigarh, IND; 5 Neurology, Postgraduate Institute of Medical Education and Research, Chandigarh, IND

**Keywords:** covid-19, acute respiratory distress syndrome (ards), high-dose dexamethasone, fungal infection, secondary infection, pulse dose steroids, tocilizumab, cytokine storms

## Abstract

Background and objectives

Recent randomized controlled trials (RCTs) have indicated potential therapeutic benefits with high-dose dexamethasone (HDD) or tocilizumab (TCZ) plus standard care in moderate to severe coronavirus disease 2019 (COVID-19) with acute respiratory distress syndrome (ARDS). No study has compared these two against each other. We aimed to compare the efficacy and safety of HDD against TCZ in moderate to severe COVID-ARDS.

Methods

Patients admitted with moderate to severe COVID-19 ARDS with clinical worsening within 48 hours of standard care were randomly assigned to receive either HDD or TCZ plus standard care. The primary outcome was ventilator-free days (VFDs) at 28 days. The main secondary outcomes were 28-day all-cause mortality and the incidence of adverse events. Our initial plan was to perform an interim analysis of the first 42 patients.

Results

VFDs were significantly lower in the HDD arm (median difference: 28 days; 95% confidence interval (CI): 19.35-36.65; Cohen’s d = 1.14;p* *< 0.001). We stopped the trial at the first interim analysis due to high 28-day mortality in the HDD arm (relative risk (RR) of death: 6.5;* *p = 0.007; NNT (harm) = 1.91). The incidence of secondary infections was also significantly high in the HDD arm (RR: 5.5; p = 0.015; NNT (harm) = 2.33).

Conclusions

In our study population, HDD was associated with a very high rate of mortality and adverse events. We would not recommend HDD to mitigate the cytokine storm in moderate to severe COVID-19 ARDS. TCZ appears to be a much better and safer alternative.

## Introduction

Coronavirus disease 2019 (COVID-19) has been associated with high mortality in moderate and severe acute respiratory distress syndrome (ARDS). The hyper-inflammatory response triggered by SARS-CoV-2 is characterized by the overproduction of pro-inflammatory cytokines, leading to organ dysfunction [1,2]. Intervening timely with immunomodulatory therapies might mitigate the severity. Based on this assumption, researchers have been focusing on several interventions, including IL-6 inhibitors and corticosteroids [3-10]. The largest trial of tocilizumab (TCZ) to date has shown significant survival benefits with TCZ plus standard care [11]. However, the dose of corticosteroids in COVID-19 has remained controversial. Although the results of two recent randomized controlled trials (RCTs) have shown potential therapeutic benefits with dexamethasone, the practice remains variable [12,13].

The COVID-19 pandemic has exhausted the resources of low- and middle-income countries. The scenario was remarkably grim in India, amidst the second wave, with insufficient TCZ supply [14]. Affordable and widely available effective alternate immunomodulatory therapies besides TCZ were urgently needed. We hypothesized that timely treatment with high-dose dexamethasone (HDD) may downregulate the integrated pathways of inflammation-coagulation-fibroproliferation and potentially improve patient outcomes. To the best of our understanding, this is the first RCT to compare the efficacy of HDD against TCZ in patients with moderate to severe COVID-19 ARDS.

## Materials and methods

Study design

This study was conducted between May 6 and June 28, 2021, at a tertiary care hospital in India. Our objective was to investigate the efficacy and safety of early rescue therapy with HDD versus TCZ in COVID-19 unresponsive to standard care. The study protocol, statistical analysis proposal, and criteria for premature study termination were planned a priori (Figure 4 and Figure 5 in the Appendices). The trial was approved by the Institutional Ethics Committee (reference number: NK/7349/Study/939) and registered in the clinical trial registry of India (CTRI/2021/04/033263 (April 30, 2021)). Prior to enrolment and randomization, written informed consent was taken from the participants or their legal representatives.

Participants

Participants aged 18 years and older, with confirmed SARS-CoV-2 infection by reverse-transcriptase polymerase chain reaction (RT-PCR) assay, were recruited. Patients with a partial pressure of arterial oxygen to fraction of inspired oxygen (PaO_2_/FiO_2_) ratio of less than 200 on admission and receiving standard care were screened for eligibility. Among these patients, those with clinical worsening in less than 48 hours of the initiation of standard care were randomized. Clinical worsening was defined as follows: (1) decrease in PaO_2_/FiO_2_ by more than 50 of the baseline admission value, (2) oxygenation/ventilation device is upgraded, and (3) static or rising levels of C-reactive protein (CRP > 50 mg/L).

The exclusion criteria included patients with prior history of immunosuppression and use of immunosuppressive drugs, raised septic biomarkers suggestive of invasive bacterial or fungal infection, AST/ALT ≥ five times the upper limit of normal, leukocytes < 2 × 10^3^/μL, thrombocytes < 50 × 10^3^/μL, and acute or chronic diverticulitis.

Randomization

Block randomization was done using an online random number generator with varying block sizes with the unique subject or patient code generated against the block sequence number [15]. This is an open-label study, and after randomization, there was no masking. The investigators, treating clinical teams, and participants were not blinded, whereas the research personnel compiling and analyzing the outcome data were blinded to the group allotment.

Procedure

Patients with COVID-19 who were admitted to our hospital with PaO_2_/FiO_2_ < 200 received standard care as per the hospital treatment protocol. Standard care included (a) oxygen supplementation; (b) intravenous (i.v.) remdesivir loading dose of 200 mg on day 1, followed by 100 mg for the next four days; (c) i.v. dexamethasone 6 mg for 10 days; (d) therapeutic low-molecular-weight heparin 1.5 mg/kg/day; and (e) proning.

Within 48 hours of the initiation of standard care, if a patient showed clinical worsening, they were randomized to one of the intervention arms, HDD or TCZ. Patients in the HDD arm received i.v. dexamethasone 20 mg once daily for three days plus standard care until day 10. HDD dose of 20 mg was selected on the basis of a recent RCT [13]. Patients in the TCZ arm received a single i.v. infusion of TCZ 6 mg/kg plus standard care of 6 mg dexamethasone for 10 days. An additional dose of TCZ (6 mg/kg) will be administered if the patient shows no clinical improvement within 24 hours. The low dosing of TCZ was based on a previous study and due to supply considerations [14,16].

Outcomes

The primary outcome was ventilator-free days (VFDs) within 28 days since randomization. The secondary endpoints were all-cause mortality, the incidence of adverse events (i.e., secondary infections, insulin requirement for hyperglycemia, and vasopressor requirement), variation in the Sequential Organ Failure Assessment (SOFA) score and WHO Clinical Progression Scale (WHO-CPS), duration of ICU stay, CRP variation, time to negative result on RT-PCR, and time to discharge.

Statistical analysis

The study was designed to compare the means of VFDs across the two groups to demonstrate an effect size of 0.8 (Cohen’s d) with a power of 80% and α at 0.05. A sample size of 42 across two groups (21 per group) was estimated using G power 3.1.9.4. A priori, when this sample size had been reached, we planned for an interim analysis to decide whether to proceed with recruitment to our secondary sample size or to stop the trial based on preset criteria (Figure 5 in the Appendices). Normality was assessed using skewness indicators and/or Q-Q plots. Categorical data have been expressed as count (%) and were analyzed using the Chi-squared/Fisher’s exact test. Time to event analysis has been done using the Kaplan-Meier (K-M) survival estimates, competing risks regression, and Cox proportional hazards, where assumptions have been met and the model fit was significantly better than the null. Missing data was less than 5%, so no imputation methods were used. The analysis has been done using SPSS version 25.0 for Windows (SPSS Inc., Chicago, IL, USA), Stata 15 (StataCorp. 2017. Stata Statistical Software: Release 15. College Station, TX: StataCorp LLC), and R studio 1.4.1130.0.

## Results

**Figure 1:**
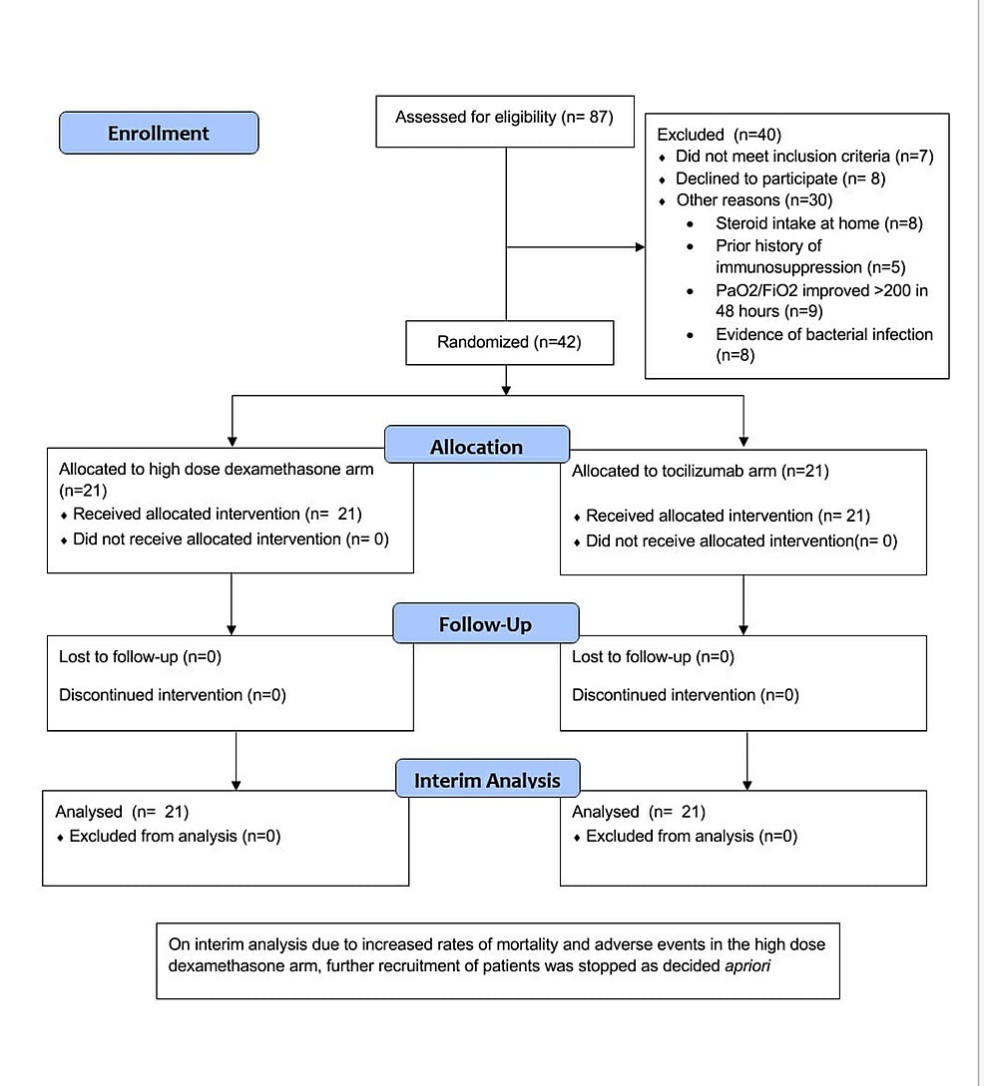
Consolidated Standards of Reporting Trials (CONSORT) flow diagram of the study.

A total of 87 patients with COVID-19 ARDS on admission were screened for inclusion, of whom 42 were randomized (Figure 1). The demography, clinical characteristics, and biomarkers of the patients at baseline and intervention are presented in Table 1.

**Table 1:** Demography, clinical characteristics, and biomarkers of patients at baseline and intervention. *p-value < 0.05 was considered significant; a: Mann–Whitney U-test; b: Chi-squared/Fisher’s exact test. IQR: interquartile range; PaO2/FiO2: partial pressure of arterial oxygen to fraction of inspired oxygen.

		High-dose dexamethasone arm (n = 21)	Tocilizumab arm (n = 21)	p-value
Age, median (IQR), years		51 (45–58)	50 (44–65)	0.920^a^
BMI, median (IQR), kg/m^2^		30.20 (26.4–35.6)	27.45 (25.90–30.61)	0.232^a^
Sex, number (%)	Male	12 (57.14%)	12 (57.14%)	1.000^b^
	Female	9 (42.86%)	9 (42.86%)	
Coexisting conditions, number (%)	Diabetes mellitus	7 (33.33%)	8 (38.10%)	0.747^b^
	Hypertension	11 (52.38%)	13 (61.90%)	0.533^b^
	Chronic kidney disease	0 (0%)	0 (0%)	-
	Coronary artery disease	0 (0%)	1 (4.76%)	1.000^b^
	Chronic liver disease	0 (0%)	0 (0%)	-
	Chronic obstructive pulmonary disease	0 (0%)	2 (9.52%)	1.000^b^
	Asthma	0 (0%)	1 (4.76%)	1.000^b^
	Hypothyroid	2 (9.52%)	1 (4.76%)	1.000^b^
	Pregnancy	1 (4.76%)	2 (9.52%)	0.698^b^
Days from symptom onset, median (IQR), days	On admission	6 (6–7)	7 (6–7)	0.039^a^
	On the first dose of intervention (high-dose dexamethasone or tocilizumab)	7 (7–8)	8 (7–9)	0.011*^a^
PaO_2_/FiO_2_, median (IQR), mmHg	On admission	125.14 (110.29–138.67)	134.5 (117–181)	0.07^a^
	On the first dose of intervention (high-dose dexamethasone or tocilizumab)	81.07 (68.22–91.60)	80.93 (62.20–113)	0.920^a^
Respiratory support at admission, number (%)	Invasive mechanical ventilation	0 (0%)	0 (0%)	0.617^b^
	Noninvasive ventilation	2 (9.52%)	1 (4.76%)	
	High-flow nasal cannula	1 (4.76%)	2 (9.52%)	
	Non-rebreather mask	15 (71.43%)	12 (57.14%)	
	Face mask/nasal prongs	3 (14.29%)	6 (28.57%)	
Respiratory support at intervention, number (%)	Invasive mechanical ventilation	1 (4.76%)	1 (4.76%)	0.597^b^
	Noninvasive ventilation	8 (38.10%)	5 (23.81%)	
	High-flow nasal cannula	12 (57.14%)	15 (71.43%)	
	Non-rebreather mask	0 (0%)	0 (0%)	
	Face mask/nasal prongs	0 (0%)	0 (0%)	
Hours in prone position during hospital stay, median (IQR)		96 (0–128)	48 (0–80)	0.063^a^
Laboratory variables at admission, median (IQR)	C-Reactive protein, median (IQR), mg/dL	54.2 (33.4–75.1)	75 (47–90)	0.218^a^
	White blood cell count, median (IQR), × 10^3^/μL	8.4 (7.6–10.7)	8.1 (7.30–9.70)	0.413^a^
	Neutrophil/lymphocyte ratio	15.62 (12.21–20.75)	16.40 (10.33–21.40)	0.811^a^
	Platelet count, × 10^3^/μL	246 (189–316.5)	221 (168–276 )	0.083^a^
	Ferritin, ng/mL	702.5 (503.2–989)	522 (321.8–969)	0.252^a^
	D-Dimer, ng/mL	1568 (694–3455)	853 (512–2388)	0.204^a^
Laboratory variables at intervention, median (IQR)	C-Reactive protein, median (IQR), mg/dL	89.2 (72–135.70)	111 (74.30–151.40)	0.443^a^
	White blood cell count, median (IQR), × 10^3^/μL	11.2 (9.3–13.20)	10.6 (9.1–11.70)	0.227^a^
	Neutrophil/lymphocyte ratio	17 (10.94–21.78)	13 (9.73–19.10)	0.489^a^
	Platelet count, median (IQR), × 10^3^/μL	233 (196–347.5)	258 (172–357)	0.597^a^
	Ferritin, median (IQR), ng/mL	607 (428.45–1410)	631.9 (256.65–992.77)	0.170^a^
	D-Dimer, median (IQR), ng/mL	1118 (541.65–3513.1)	649 (389.38–1734.75)	0.930^a^

Univariate analysis

Primary Outcome

**Figure 2:**
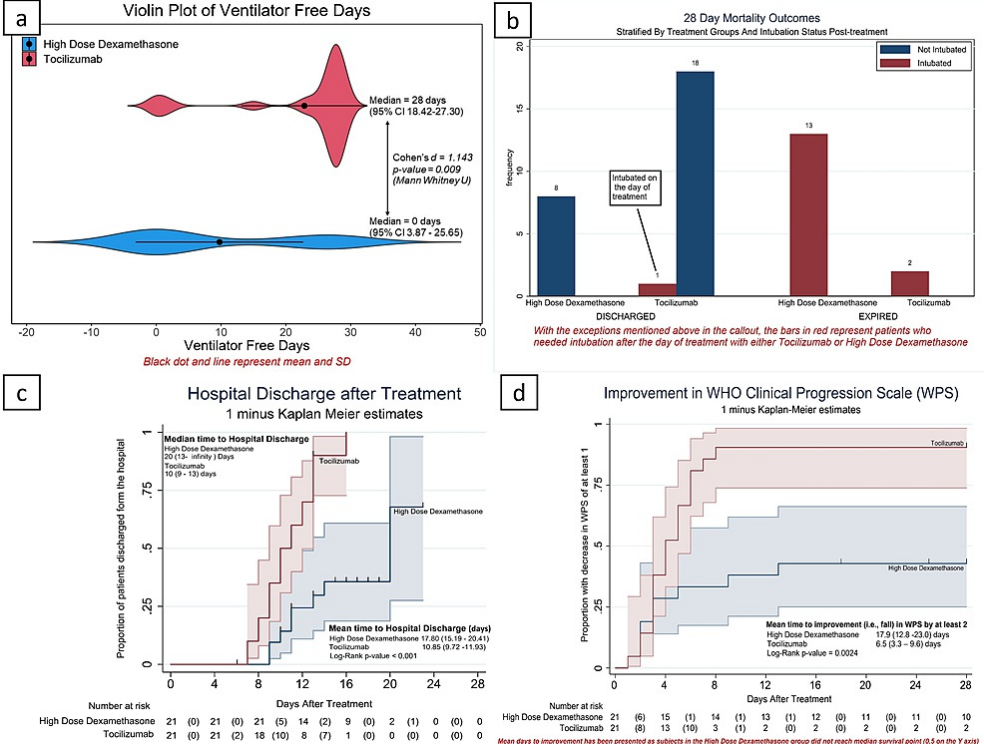
Outcomes. (a) Violin plot of ventilator-free days. (b) Bar diagram showing mortality distributed among the treatment group and posttreatment intubation status. One patient was intubated on the day of therapy in the tocilizumab arm and was successfully extubated as well. (c) Kaplan–Meier estimates of cumulative hospital discharge rates and (d) improvement in WHO Clinical Progression Scale.

VFDs were significantly lower in the HDD group (9.76 ± 12.94 (95% CI: 3.87-25.65) versus 22.86 ± 9.75 (95% CI: 18.42-27.30); Cohen’s d = 1.14; p < 0.001) at a calculated power of 99.99% (Figure 2a). The median difference was 28 days (95% CI: 19.35-36.65) (Table 2).

**Table 2:** Outcomes. *p-value < 0.05 was considered significant; a: Mann–Whitney U-test; b: Chi-squared/Fisher's exact test; c: log-rank test from Kaplan–Meier survival estimates (see text and Appendices for further details). HDD: high-dose dexamethasone; TCZ: tocilizumab; CI: confidence interval; IQR: interquartile range; MV: mechanical ventilation; ICU: intensive care unit; SOFA: Sequential Organ Failure Assessment score; WHO-CPS: World Health Organization Clinical Progression Scale; RT-PCR: reverse-transcriptase polymerase chain reaction.

Outcomes		HDD arm (n = 21)	TCZ arm (n = 21)	p-value
Primary outcome				
Ventilator-free days	Mean ± SD (95% CI)	9.76 ± 12.94 (3.87–25.65)	22.86 ± 9.75 (18.42–27.30)	
	Median (IQR)	0 (0–25)	28 (24–28)	0.001*^a^
Secondary outcome				
28-Day results	All-cause mortality, number (%)	13 (61.90%)	2 (9.52%)	<0.001*^b^
	Intubation rates posttreatment, number (%)	13 (61.90%)	2 (9.52%)	<0.001*^b^
	ICU free, median (IQR), days	1 (1–5)	4 (3.5–5.5)	0.017*^a^
	MV duration, median (IQR), days	12 (2.5–15.5)	0 (0–3)	<0.001*^a^
Discharged from the hospital within 28 days, number (%)		8 (38.10%)	19 (90.48%)	0.030*^b^
SOFA score, median, (IQR)	On treatment day	5 (4–8)	5 (4–6)	0.353^a^
	48 hours later	4 (4–8)	4 (4–5)	0.303^a^
	7 days after intervention	5 (2–7)	2 (2–2)	0.002*^a^
WHO-CPS score, median, (IQR)	On treatment day	6 (6–6)	6 (6–6)	0.573^a^
	7 days after intervention	6 (5–8)	5 (3–5)	<0.001*^a^
Mean time (days) to improvement in WHO-CPS score by 1 (i.e., a decrease by 1)		17.90 (underestimated)	6.48 (underestimated)	0.002*^c^
Renal replacement therapy, number (%)		2 (9.52%)	0 (0%)	0.488^b^
Vasopressor use, number (%)		13 (61.90%)	3 (14.29%)	0.001*^b^
Time to RT-PCR negative status (days), median (IQR)		19 (17–19)	17 (16–17)	0.026*^a^
Hospital stay, median (IQR), days		17 (13–17)	12 (11–12)	0.003*^a^

Secondary Outcomes

All-cause mortality at 28 days was significantly higher at 61.9% (95% CI: 39.06%-80.46%) in the HDD group, compared with 9.52% (95% CI: 2.21%-32.89%) in the TCZ group, with a p < 0.001, a large effect size of w = 0.72, and calculated power > 97% (Figure 2b). The relative risk (RR) of death in the HDD group was 6.5 (95% CI: 1.67-25.33; p = 0.007; NNT (harm) = 1.91). The preventable fraction for mortality in the TCZ group was computed as 0.79 (95% CI: 0.064-0.98) with a preventable fraction in the population of 0.333. The proportion of patients discharged at day 28 was significantly higher in the TCZ group at 90.48% (95% CI: 67.1%-97.79%) versus 38.10% (95% CI: 19.54%-60.93%) in the HDD group (Table 2).

The SOFA and WHO-CPS scores were significantly better in the TCZ group on day 7 after the intervention, paralleling an improvement in the PaO_2_/FiO_2_ ratio on day 7 in the TCZ group (median difference: 132.96 (95% CI: 55.15-210.77; p < 0.001)) (Figure 6 in the Appendices). The proportion of patients requiring vasopressors was 61.90% in the HDD group against 14.29% in the TCZ group (p = 0.001). The median number of days a patient remained RT-PCR positive for SARS-CoV-2 was higher in the HDD group. The duration of hospital stay was also high in the HDD group (Table 2).

**Figure 3:**
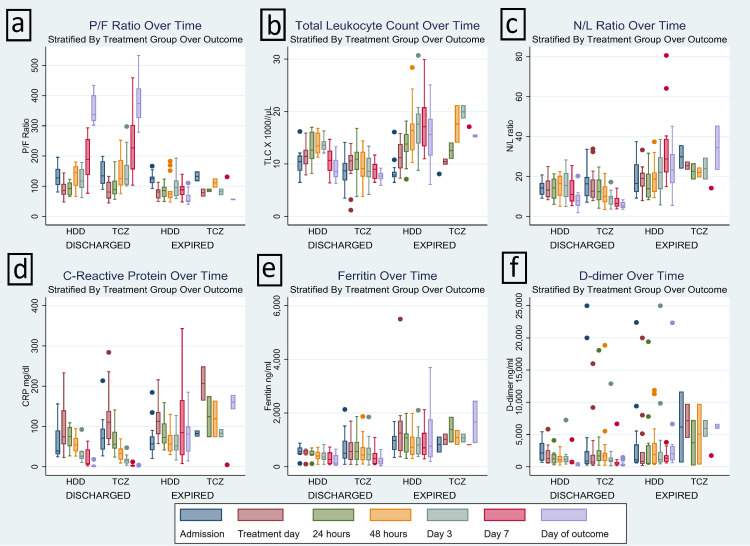
Boxplots of biomarkers stratified by treatment groups and outcome (discharged/expired) at various time points: (a) PaO2/FiO2 ratio (PFR), (b) total leukocyte count (TLC), (c) neutrophil/lymphocyte ratio (N/L ratio or NLR), (d) C-reactive protein (CRP), (e) D-dimer, and (f) ferritin.

**Figure 4:**
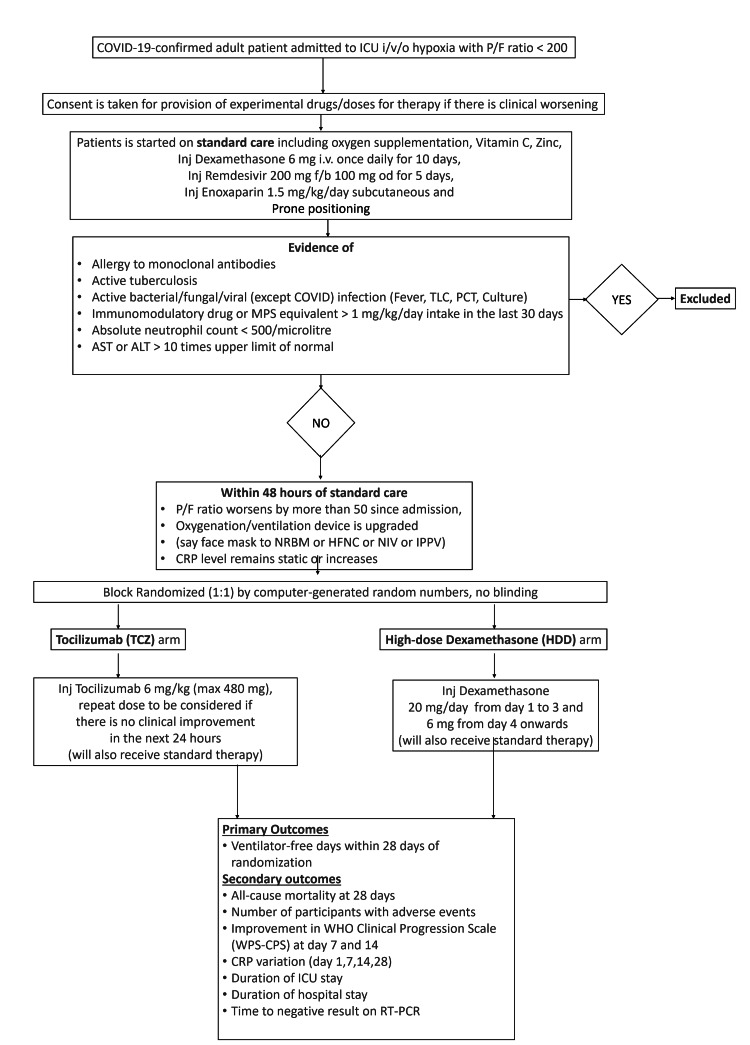
The proposed study protocol. PaO_2_/FiO_2_: partial pressure of arterial oxygen to fraction of inspired oxygen; f/b: followed by; MPS: methylprednisolone; TLC: total leucocyte count; AST: aspartate aminotransferase; ALT: alanine aminotransferase; CRP: C-reactive protein; WHO-CPS: World Health Organization Clinical Progression Scale; NRBM: non-rebreather mask; HFNC: high-flow nasal cannula; NIV: noninvasive ventilation; IPPV: invasive positive pressure ventilation.

**Figure 5:**
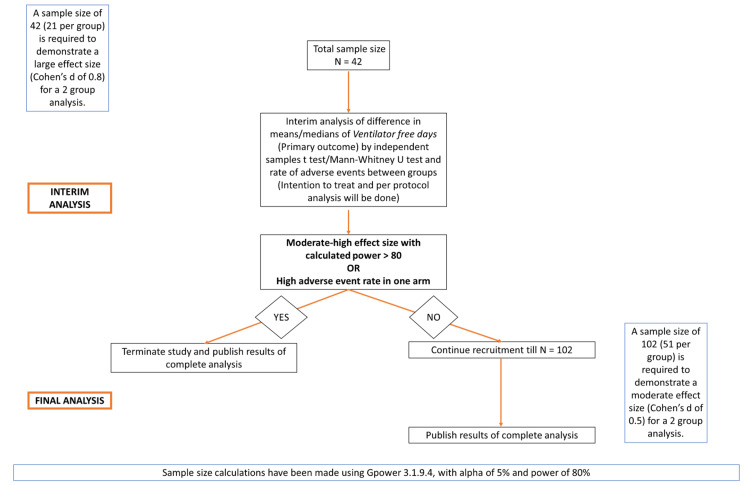
Statistical analysis/interim analysis plan.

**Figure 6:**
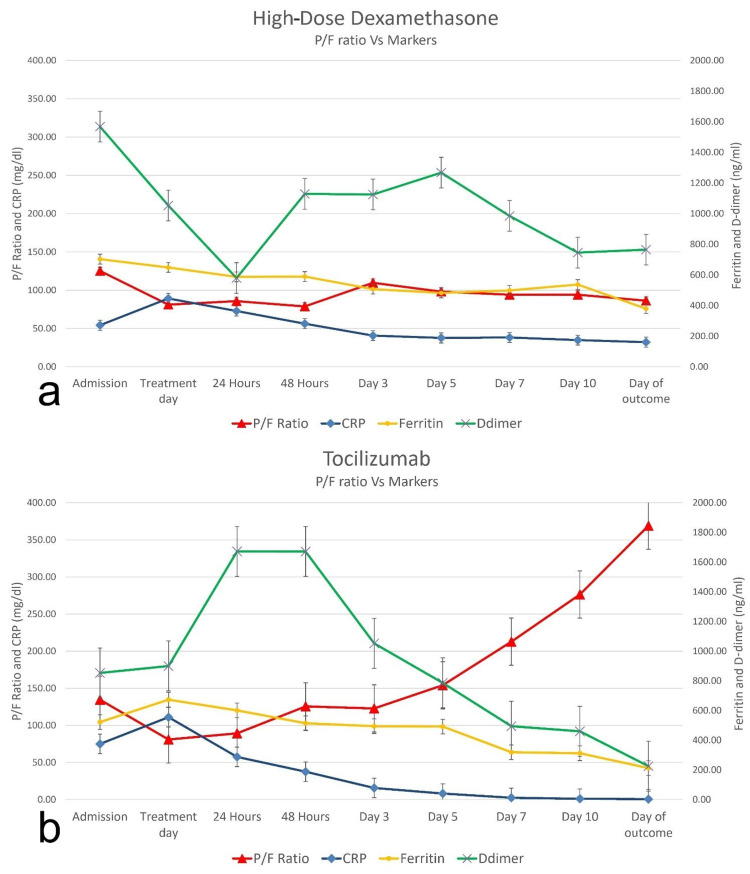
Trend of PaO2/FiO2 (P/F) ratio with biomarkers in each group (median values and standard errors have been plotted).

**Figure 7:**
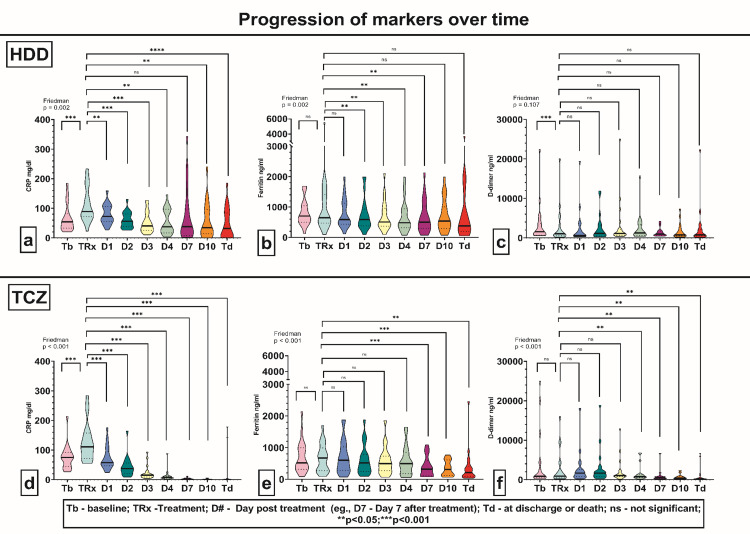
Panel of violin plots showing progression over time of CRP (a and d), ferritin (b and e), and D-dimer (c and f): a, b, and c for the high-dose dexamethasone (HDD) group, and d, e, and f for the tocilizumab (TCZ) group. Pairwise comparisons are with the Wilcoxon signed-rank test. CRP: C-reactive protein.

**Figure 8:**
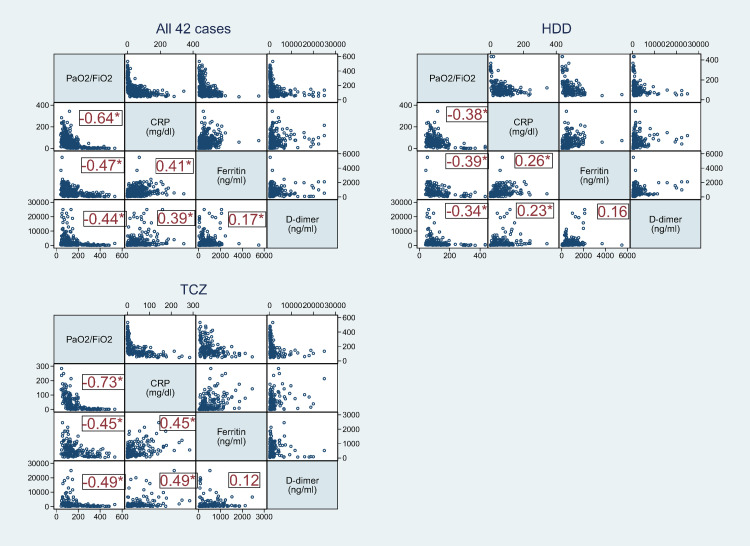
Scattergraph showing correlations between PaO2/FiO2 ratio, CRP, ferritin, and D-dimer in all cases and by groups. HDD: high-dose dexamethasone; TCZ: tocilizumab; CRP: C-reactive protein; non-axial numbers (maroon): Spearman rank correlation coefficients; *: p-value < 0.05.

The distributions of PaO_2_/FiO_2_, total leucocyte count (TLC), neutrophil/lymphocyte (N/L) ratio, CRP, ferritin, and D-dimer in both groups at various time points are presented in Figure 3 (also see Table 4, Figure 6, and Figure 7 in the Appendices). CRP had the best negative correlation with PaO_2_/FiO_2_ (Figure 8 in the Appendices).

Multivariate and survival analysis

The median time to discharge was 20 days (95% CI: 13 to infinity) in the HDD group against 10 days (95% CI: 9-13) with a log-rank test p-value < 0.001 (Figure 2c). The median time to RT-PCR negative status was 12 days (95% CI: 11-14) in the HDD group and 10 days (95% CI: 9-10) in the TCZ group (log-rank test p = 0.006). A K-M analysis with a similarly censored time variable and WHO-CPS improvement as the dependent variable gave a mean time to the improvement of 17.9 (95% CI: 12.80-23.00) in the HDD group against 6.48 (95% CI: 3.40-9.55) in the TCZ group (Figure 2d and Table 5 in the Appendices).

After assessing for proportionality, the Cox proportional hazards model was fit on the above and adjusted for the variables PaO_2_/FiO_2_ ratio at baseline, days from symptom onset at intervention, CRP, TLC, and N/L ratio at intervention. This gave a hazard ratio of 3.69 (95% CI: 1.34-10.15; p = 0.024) for WHO-CPS improvement in the TCZ group (Figure 2d and Table 6 in the Appendices).

**Figure 9:**
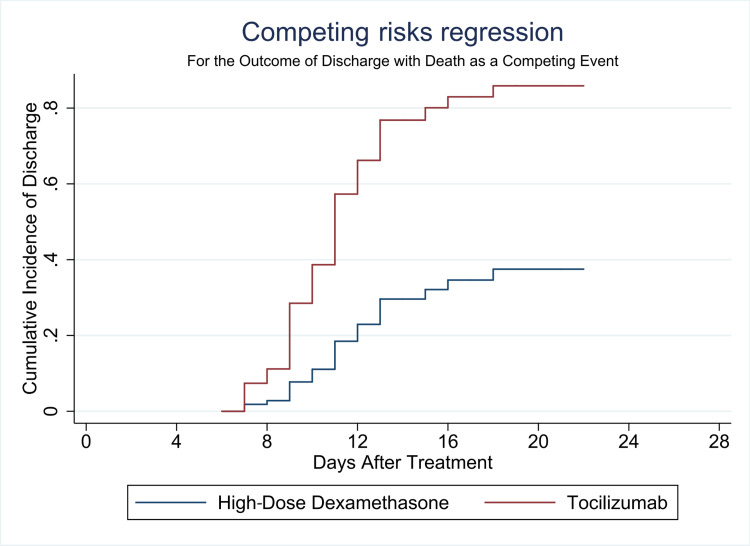
Competing risks regression curves for the outcome of discharge.

**Figure 10:**
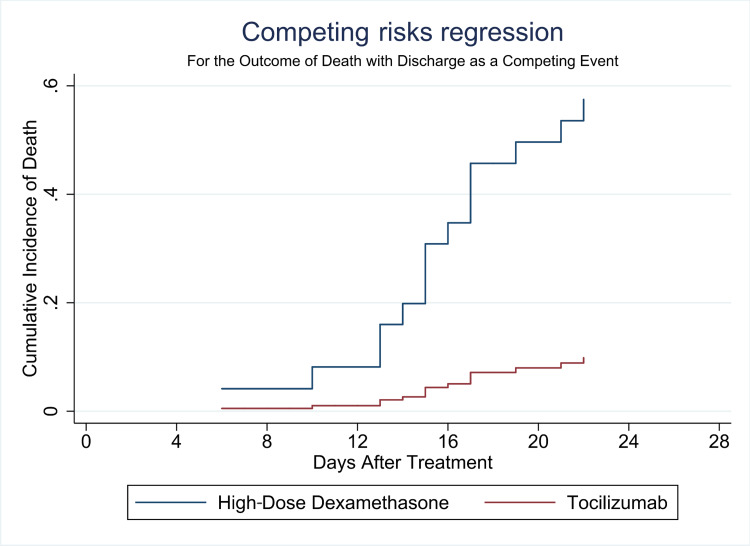
Competing risks regression curves for the outcome of death.

A competing risks regression with days posttreatment as the time variable and death as competing interest gave an adjusted sub-hazard ratio (SHR) for discharge of 5.86 (95% CI: 1.49-23.04; p = 0.011) in the TCZ group. Similarly, with discharge as a competing interest, the TCZ group had an adjusted SHR for death of 0.085 (95% CI: 0.016-0.44; p = 0.003) (Table 7, Figure 9, and Figure 10 in the Appendices).

Adverse event outcomes

The main reason the trial was stopped at the interim analysis stage was the increased mortality and adverse event rate observed in the HDD arm. This was chiefly due to new infections in HDD (relative risk: 5.5; 95% CI: 1.38-21.86; p = 0.015; NNT (harm) = 2.33; 95% CI: 5.53-1.48). Table 3 summarizes the adverse events per Common Terminology Criteria for Adverse Events (CTCAE) version 5 [17]. The overall adverse event rate per 100 patient days was 61.43 in the HDD group versus 27.72 in the TCZ group.

**Table 3:** Adverse events. All cardiac arrests were grade 5. ‡Excludes deaths and infections to avoid duplication. ᴪBlood sugar > 180 mg/dL. *p-value < 0.05 was considered significant. HDD: high-dose dexamethasone; TCZ: tocilizumab; CTCAE: Common Terminology Criteria for Adverse Events.

		Adverse events, number of patients (%)			Number of events		Event rate ratio HDD/TCZ	p-value (exact rate ratio test)
		Total (n = 42)	HDD (n = 21)	TCZ (n = 21)	HDD (number in 306 patient days)	TCZ (number in 220 patient days)		
Deaths		15 (36.06%)	13 (61.9%)	2 (9.52%)	13	2	4.67 (1.06–42.65)	0.023*
Infections		13 (30.95%)	11 (52.38%)	2 (9.52%)	25	2	8.9 (2.24–78.28)	<0.001*
Grade 3 or worse adverse events by CTCAE version 5, MedDRA system organ class preferred terms								
Cardiac disorders	Supraventricular tachycardia	1 (2.38%)	0 (0%)	1 (4.76%)	0	1	0 (0–28.04)	0.41
	Sinus bradycardia	1 (2.38%)	0 (0%)	1 (4.76%)	0	1	0 (0–28.04)	0.41
	Cardiac arrest	15 (36.06%)	13 (61.9%)	2 (9.52%)	13	2	4.67 (1.06–42.65)	0.023*
Infections or infestations	Fungemia	8 (19.04%)	8 (38.09%)	0 (0%)	8	0	Zero denominator	0.013*
	Catheter-related infection	7(16%)	6 (28.27%)	1 (4.76%)	6	1	4.31 (0.52–198.42)	0.158
	Lung infection	11 (26.19%)	10 (47.61%)	1 (4.76%)	11	1	7.91 (1.15–340.41)	0.016*
Respiratory, thoracic, and mediastinal disorders	Grade 4 adult respiratory distress syndrome	15 (36.06%)	13 (61.9%)	3 (14.28%)	13	3	3.12 (0.85–17.04)	0.065
Vascular disorders	Shock	16 (38.09%)	13 (61.90%)	3 (14.29%)	13	3	3.12 (0.85–17.04)	0.065
Metabolism and nutrition disorders	Hyperglycemia^ᴪ^	30 (71.43%)	21 (100%)	9 (42.86%)	106	49	1.56 (1.09–2.23)	0.009*
Gastrointestinal disorders	Gastric hemorrhage	4 (9.52%)	4 (19.04%)	0 (0%)	18	0	Zero denominator	<0.001*
Total number of events^‡^					188	61	2.22 (1.65–3.01)	<0.001*

## Discussion

Steroids have been extensively used and evaluated since the beginning of the pandemic. Several cohort studies described varied findings, either favorable or unfavorable, promoting confusion especially when it concerns the dose of steroids [8-10]. The first RCT on the role of steroids in COVID-19 has recommended that dexamethasone 6 mg once daily for 10 days decreased mortality [12]. A recent RCT on HDD has shown therapeutic benefit at doses of 20 mg per day in critically ill patients with COVID-19 [13]. Treatment with HDD was beneficial in lowering mortality and the period of mechanical ventilation in critically ill patients with non-COVID-19 ARDS [18]. Despite these promising results, there is still uncertainty regarding the role of HDD in COVID-19. Several meta-analyses have claimed TCZ to be a safe and effective drug in reducing the risk of death [19-21]. In a low- to middle-income country with scarce TCZ supply amidst the pandemic, we surmised that HDD would be an easily accessible, low-cost, and potentially effective treatment option. At moderate or high doses, it has not been linked with detrimental effects [12,13]. Hence, we sought to compare the therapeutic effectiveness of HDD and TCZ in COVID-19.

VFDs were selected as the principal outcome as it takes into account mortality and the period of ventilation together in a manner that summarizes the net effect of an intervention on these parameters [22]. The major difference between recent RCTs and our study is that patients with clinical worsening within 48 hours of receiving standard care were treated with HDD or TCZ, as a rescue, second-line therapy [11-13,19-21]. Characteristically, ARDS presents with a profound pulmonary and systemic inflammatory reaction within 48 hours, giving rise to aggravated pulmonary inflammation and fibroproliferation [23]. Failed efforts to halt the self-perpetuating tissue inflammation within a specified time lead to the subsequent suppression of lung function and increased chances of mortality. Therefore, we ensured that all the randomized patients unresponsive to standard care received immediate rescue therapy within 48 hours of worsening ARDS.

Notably, our findings show that HDD was associated with high 28-day mortality and was poorly tolerated. There was a significantly higher incidence of adverse events, especially new infections. This high incidence was beyond the predetermined limits of futility, fostering a very weak probability of a large trial. Our findings have unveiled the ineffectiveness and poor safety of HDD therapy in COVID-19 ARDS with PaO_2_/FiO_2 _< 200. Hence, as decided by the institute clinical management board, the trial was stopped immediately after the prespecified interim analysis.

Our study results favor the use of TCZ in moderate to severe COVID-19. Several RCTs examining the role of TCZ in COVID-19 reported conflicting results [24,25]. These trials differed considerably in study design, illness severity of enrolled patients, and imbalances in the use of steroids between study groups. The RECOVERY trial reported all-cause mortality of 31% among patients allocated to the TCZ arm and 35% in the usual care arm (rate ratio: 0.85; 95% CI: 0.76-0.95; p = 0.0028) [11]. Our study had an all-cause mortality rate ratio of 0.21 (95% CI: 0.02-0.93; p = 0.022); however, our study was never powered to detect this outcome. Nevertheless, IL-6 inhibitors also have the potential to suppress the host immune response and could hypothetically raise the probability of acquiring secondary infections. In our trial, we did not witness a greater risk of infection or adverse events with TCZ use. These findings support previous RCTs about the safety of TCZ in COVID-19 [11,24,25].

Our study has certain limitations. First, the trial was discontinued after the first interim analysis, at a limited sample size; hence, the precision of the treatment effect estimates might be low than anticipated. However, it would be prudent to note that this interim analysis sample size was calculated to be valid for demonstrating a large effect size with adequate power when it came to VFDs as the primary outcome measure. A larger sample size, no doubt, would have been able to detect the differences in the effects of HDD or TCZ on mortality. Second, the study lacks a control arm. We did not compare outcomes against a control group that should have received only the standard care. Third, a different dose of dexamethasone might have provided a different result; therefore, the outcomes portrayed in this study should be linked only to the particular dose administered. Despite the above limitations, our robust study design and results add necessary evidence to the scientific community. Our findings await subsequent clarification from ongoing clinical trials on different doses of dexamethasone [26-28].

## Conclusions

Our study findings discourage the use of high doses of dexamethasone in the management of moderate to severe COVID-19 ARDS. The routine use of such high doses to mitigate the inflammatory cytokine storm in these patients might worsen outcomes possibly due to a high rate of secondary infections and therefore cannot be recommended.

From this study, we can conclude that tocilizumab is associated with a decreased mortality, reduced need for invasive mechanical ventilation, and a higher probability of successful hospital discharge in comparison with high-dose dexamethasone when used in the context of mitigating the adverse effects of the cytokine storm.

## Appendices

The study protocol that was initially proposed has been represented in the following flowchart (Figure 4), and the a priori statistical analysis plan is depicted in Figure 5.

The course of biomarkers is shown in Table 4 as the median difference between various time points. The relationship between the biomarkers and PaO_2_/FiO_2_ ratio is shown in Figure 6. Figure 7 depicts the median and interquartile range of the various biomarkers between groups as violin plots, and Figure 8 illustrates the correlation scatterplot between the markers and PaO_2_/FiO_2_ ratio, which shows the Spearman rank correlation coefficients. CRP appears to have the best negative correlation with the PaO_2_/FiO_2_ ratio.

**Table 4:** Course of biomarkers in our study population. *p < 0.05 was considered significant; **Mann–Whitney U-test for comparing change from baseline to treatment day and from treatment day to indicated time point between treatment groups (the negative sign indicates a decrease from each earlier mentioned time point). Median differences and 95% CIs have been derived from quantile regression. CRP: C-reactive protein.

Outcomes	High-dose dexamethasone (n = 21)	Tocilizumab (n = 21)	p-value**
CRP (mg/dL), median difference between time points (95% CI)			
Baseline to treatment day	35 (-10.88 to 80.88)	36 (-5.03 to 77.03)	0.93
Treatment day to 24 hours	-16.4 (-61.11 to 28.31)	-53.5 (-96.21 to -10.71)	0.116
Treatment day to 48 hours	-32.9 (-75.83 to 10.03)	-73.5 (-111.16 to -35.84)	0.038*
Treatment day to day 3	- 48.49 (-93.62 to -3.36)	-95.33 (-126.86 to -63.79)	0.014*
Treatment day to day 4	- 51.51 (-104.05 to 1.04)	-102.88 (-131.55 to -74.20)	0.004*
Treatment day to day 7	-51 (-111.99 to 9.99)	-108.54 (-136.85 to - 80.22)	0.001*
Treatment day to day 10	- 54.42 (-108.03 to -0.80)	-109.91 (-145.52 to -74.30)	0.002*
Treatment day to outcome day	- 57.1 (-111.02 to -3.18)	-110.36 (-137.23 to -83.48)	0.008*
Ferritin (ng/mL), median difference between time points (95% CI)			
Baseline to treatment day	-53.8 (-539.43 to 431.84)	151 (-276.89 to 578.89)	0.428
Treatment day to 24 hours	-62 (-575.75 to 451.75)	-72.1 (-543.9 to 399.7)	0.327
Treatment day to 48 hours	-59.7 (-539.28 to 419.88)	-159 (-545.61 to 227.611)	0.333
Treatment day to day 3	- 141.7 (-651.60 to 368.20)	-179 (-547.59 to 189.59)	0.428
Treatment day to day 4	- 166.7 (-671.02 to 337.62)	-114 (-1394.34 to 1166.34)	0.428
Treatment day to day 7	-150 (-693.09 to 393.09)	-333 (-705.78 to 39.78)	0.274
Treatment day to day 10	- 111.7 (-638.47 to 415.07)	-361 (-778.4 to 56.4)	0.122
Treatment day to outcome day	- 269.4 (-936.06 to 397.27)	-461 (-790.71 to -131.29)	0.333
D-Dimer (ng/mL), median difference between time points (95% CI)			
Baseline to treatment day	-514.8 (-2205.57 to 1175.97)	47 (-2053.98 to 2147.98)	0.064
Treatment day to 24 hours	-474.06 (-2062.58 to 1114.46)	772 (-809.9 to 2353.9)	0.011*
Treatment day to 48 hours	75.8 (-1513.10 to 1664.70)	771 (-901.35 to -2443.35)	0.554
Treatment day to day 3	71.8 (-1180.89 to 1324.49)	152 (-1278.13 to 1582.13)	0.155
Treatment day to day 4	214.58 (-1398.48 to 1827.64)	-114 (-1394.34 to 1166.34)	0.285
Treatment day to day 7	-68.2 (-1182.07 to 1045.67)	-357 (-1559.94 to 845.94)	0.148
Treatment day to day 10	- 307.7 (-1585.92 to 970.52)	-440 (-1979.02 to 1099.02)	0.094
Treatment day to outcome day	- 288.2 (-1639.72 to 1063.32)	-675 (-1838.18 to 488.18)	0.213

 Table 5, Table 6, and Table 7, and Figure 9 and Figure 10 show the results of the survival analysis and competing risks regression.

**Table 5:** Survival analysis outcomes (Kaplan–Meier estimates). p < 0.05 was considered significant; N.B., where median survival time could not be computed in any one group, the restricted means and extended means have been provided for careful interpretation, if necessary. HDD: high-dose dexamethasone; RT-PCR: real-time polymerase chain reaction; TCZ: tocilizumab; WPS: WHO Clinical Progression Scale.

Dependent variable	Time variable	Measured result	Values	95% CI	Statistical test	Extended mean
Discharge	Days posttreatment	Mean time HDD	17.80 (underestimated)	15.19–20.41	Log rank p value < 0.0001	24.33
		Mean time TCZ	10.85	9.72–11.93		10.85
		Median time HDD	20	13–infinity		
		Median time TCZ	10	9–13		
Expiry	Days posttreatment	Mean time HDD	16.87	15.10–18.63	Log rank p value = 0.839	16.87
		Mean time TCZ	15.05 (underestimated)	13.83–16.26		69.99
		Median time HDD	17	15–19		
		Median time TCZ	Not computed	13–infinity		
RT-PCR negative status	Days posttreatment censored at 28 days if expired	Median time HDD	12	11–14	Log-rank p-value = 0.0059	
		Median time TCZ	10	9–10		
WPS improvement by a score of 1	Days posttreatment censored at 28 days if expired	Mean time HDD	17.90 (underestimated)	12.80–23	Log-rank p-value = 0.0024	46.50
		Mean time TCZ	6.48 (underestimated)	3.40–9.55		7.61
		Median time HDD	Not computed	3–infinity		
		Median time TCZ	4	3–6		

**Table 6:** Cox proportional hazards model estimates. p < 0.05 was considered significant; #adjusting for the variables PaO_2_/FiO_2_ ratio at baseline, days from symptom onset at intervention, C-reactive protein (CRP), total leukocyte count (TLC), and neutrophil/lymphocyte (N/L) ratio at intervention. HR: hazard ratio; TCZ: tocilizumab; WPS: WHO Clinical Progression Scale.

Dependent variable	Time variable	Measured result	Values	95% CI	p-value	LR Chi-squared compared to null	Prob>Chi-squared compared to null	phtest Schonfeld residuals prob>Chi-squared (p-value)
Discharge	Days posttreatment	HR TCZ	5.153	2.090–12.702	0.001	14.93	<0.001	0.5614
		Adjusted# HR TCZ	5.106	1.628–16.009	0.005	18.38	0.053	0.5185
Expired	Days posttreatment	HR TCZ	0.843	0.159–4.471	0.841	0.04	0.839	0.5501
		Adjusted# HR TCZ	0.315	0.034–2.899	0.308	9.57	0.144	0.678
WPS improvement by a score of 1	Days posttreatment censored at 28 days if expired	HR TCZ	3.18	1.39–7.27	0.006	14.64	0.0001	0.5038
		Adjusted# HR TCZ	3.69	1.34–10.15	0.024	18.71	0.032	0.5203

**Table 7:** Competing risks regression. p < 0.05 was considered significant. HR: hazard ratio; TCZ: tocilizumab.

Dependent variable	Competing interest	Time variable	Measured result	Values	95% CI	p-value	Wald Chi-squared compared to null	Prob>Chi-squared compared to null
Discharge	Expiry	Days posttreatment	Sub-HR TCZ	4.269	1.906–9.565	<0.001	12.44	0.0004
			Adjusted# sub-HR TCZ	5.856	1.488–23.037	0.011	26.49	0.0002
Expiry	Discharge	Days posttreatment	Sub-HR TCZ	0.119	0.024–0.584	0.009	6.89	0.0087
			Adjusted# sub-HR TCZ	0.085	0.016–0.435	0.003	21.83	0.0013
